# Some Wise Men of Gotham

**Published:** 1875-08

**Authors:** 


Some Wise Men of Gotham.—Quackery in electricity is as is quackery in patent medicines, and it seems to us that physicians should be just as careful in allowing their names to be associated with the one form of rascality as with the other. Paoli's electro-voltaic belt is probably as useless a piece of furniture as any head of a family could invest his money in: patented in Washington, made in New York, it is self-puffed throughout the universe—literature, both secular and religious, from *Belgravia* to *John Wesley's Journal*, being laid under contribution to it.

Judge of our surprise, reader, when on continuing the perusal of this pamphlet, we came upon a series of recommendatory testimonials of "some of the most eminent physicians and professors of the city of New York, the originals of which can be seen at our office." The letters are really from men of position, interlarded with those from physicians, to speak mildly, not so reputable, and with those giving accounts of marvelous cures worked by these miraculous belts.

We refrain from mentioning any names, because we do not suppose these gentlemen gave these letters with a knowledge of the use to be made of them. We well remember the aid Swaim's Panacea received in a similar way in this city. The moral we desire to point is—Give us no testimonials.—*Philadelphia Medical Times.*

Salicylic Acid is soluble in three hundred parts of water and in four of alcohol. The usual way of forming an aqueous solution is to dissolve it in the smallest quantity of alcohol, and then add water to the required dilution.—*Pharmacal Gazette.*

